# Supplementary material for: Exploring Sugary Drink Consumption and Perceptions among Primary-School-Aged Children and Parents in Australia
Source: Nutrients. 2024 Sep 30;16(19):3320. doi: 10.3390/nu16193320 (PMC11478532; doi:10.3390/nu16193320)
Supplement: Supplementary file 1 [file nutrients-16-03320-s001.zip › nutrients-3163209-supplementary.pdf]

Figure S1. *Fruit Drink*

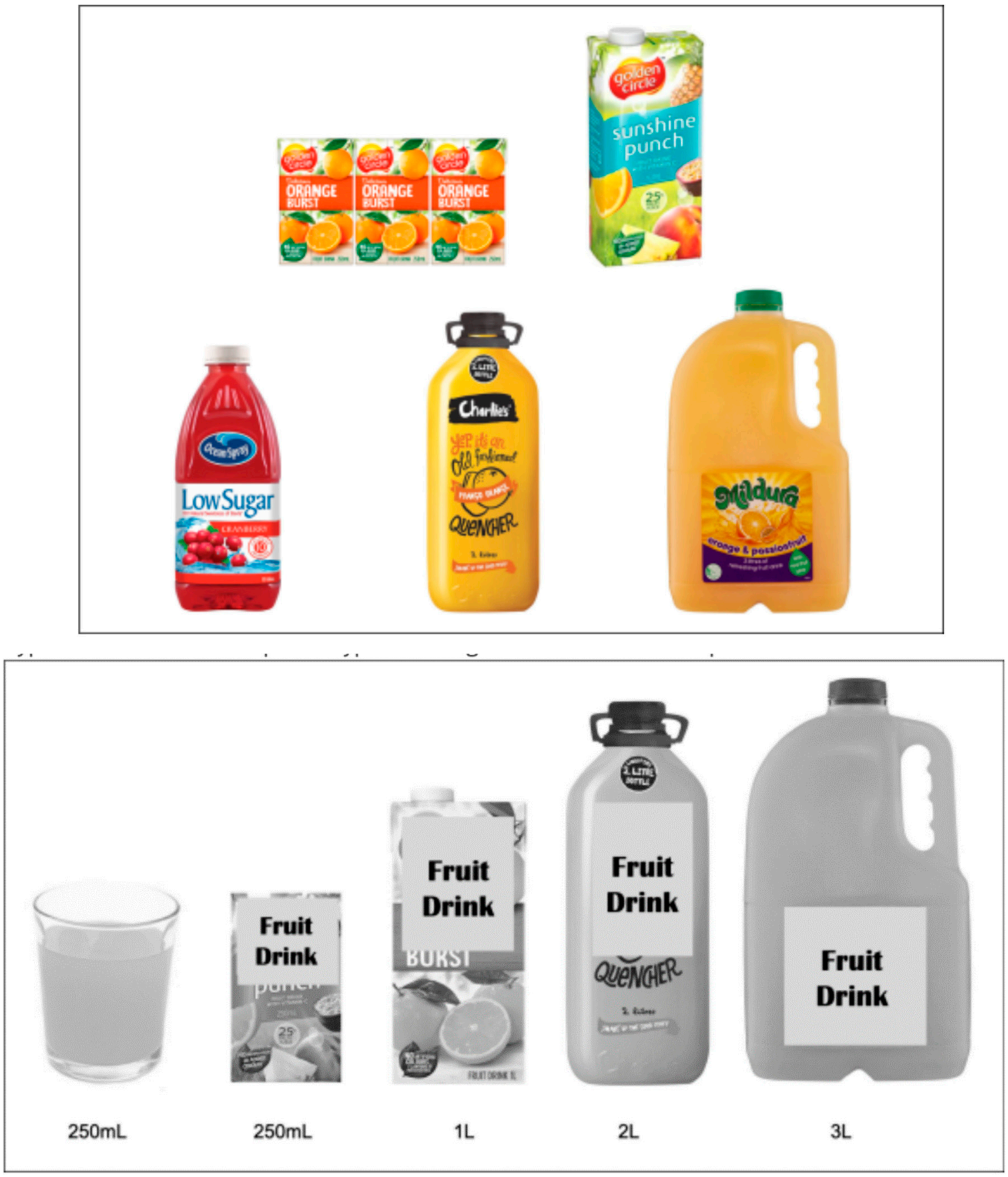

Figure SS2. *100% Fruit Juice*

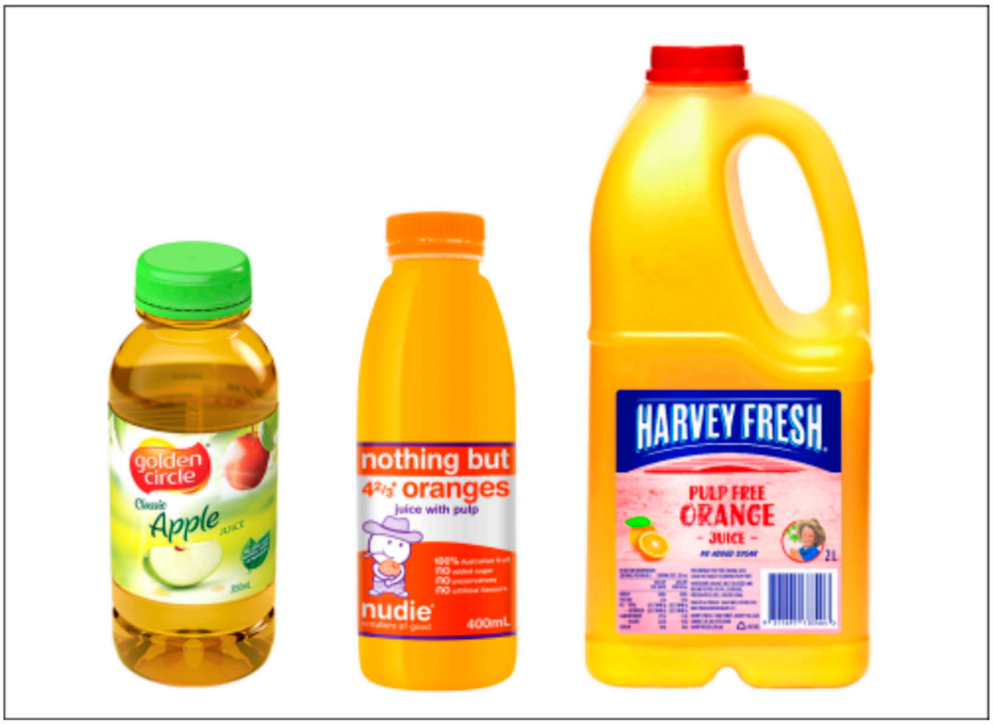

[illegible]

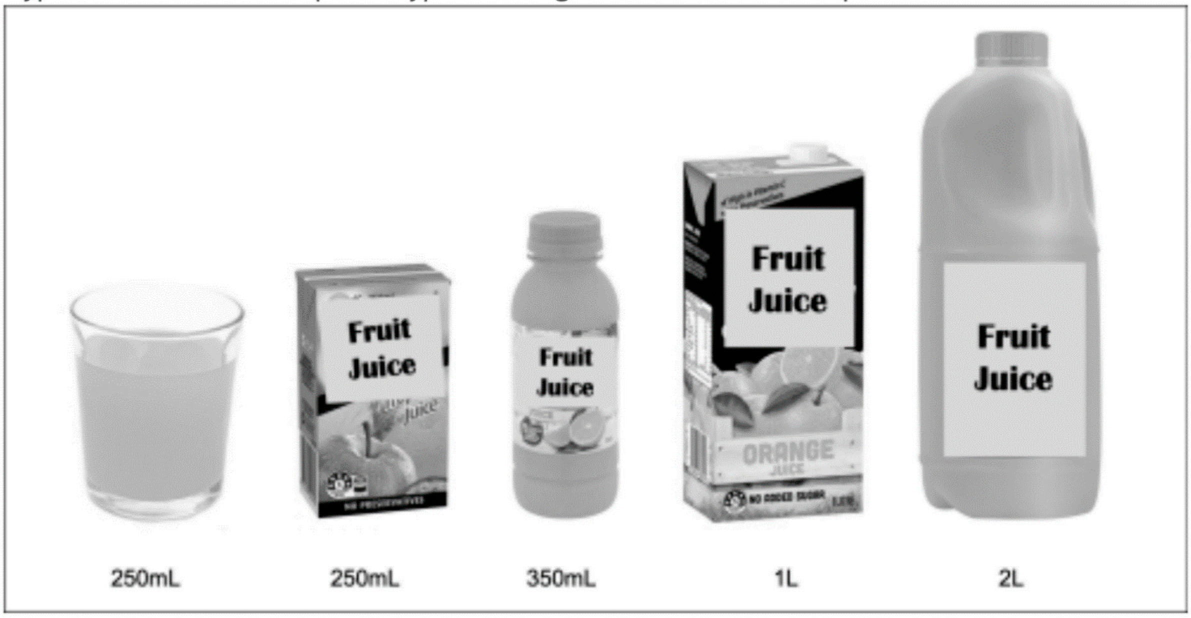

Figure S3. *Beverages containing non-nutritive sweetener* (NNS beverages)

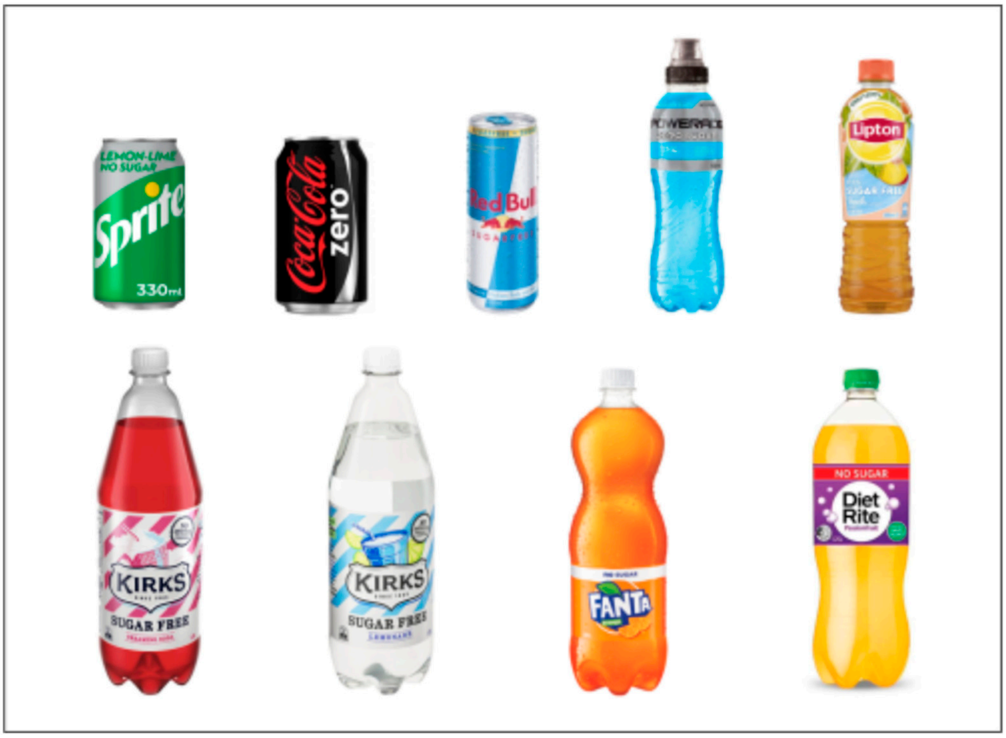

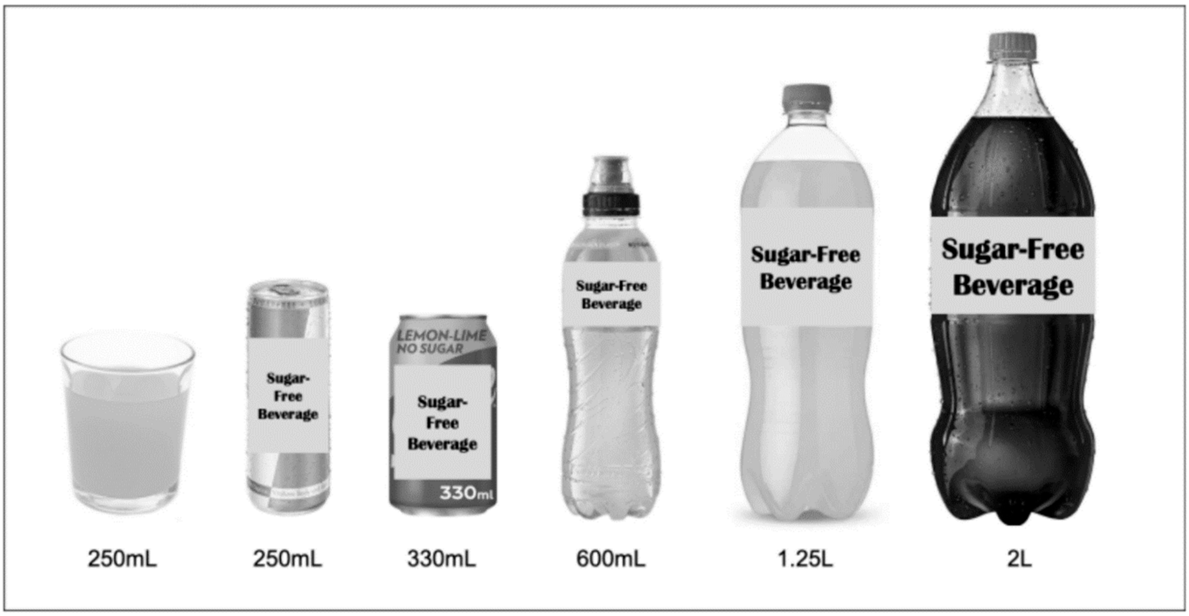

Figure S4. *Soft Drinks*

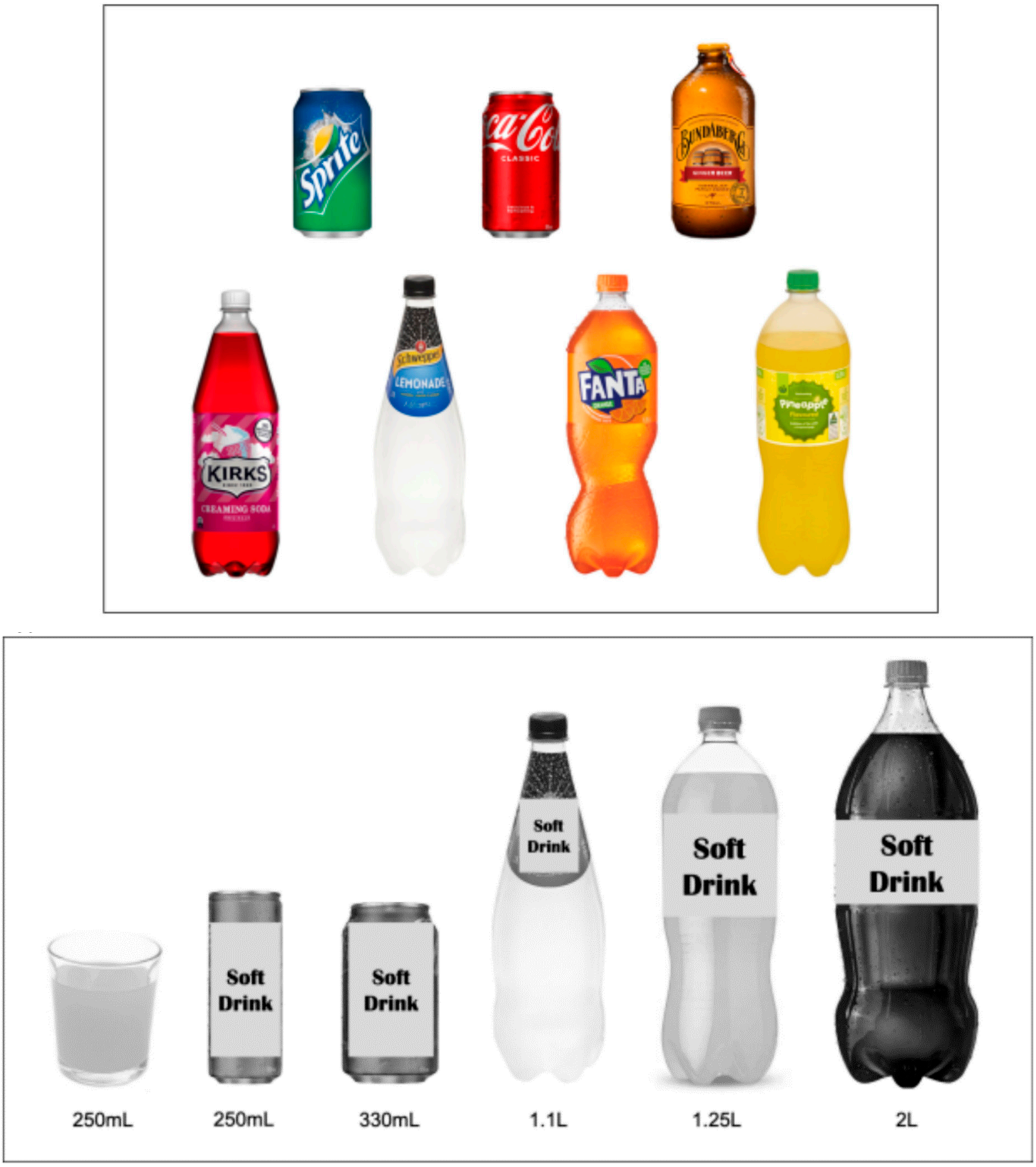

Figure S5. *Plain Milk*

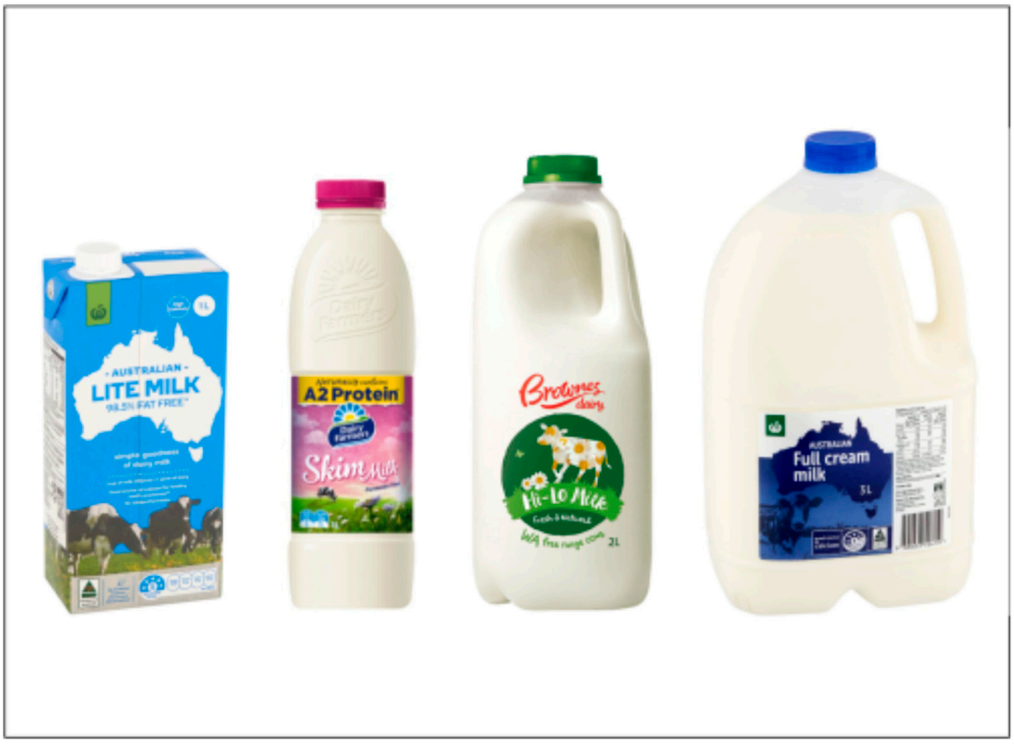

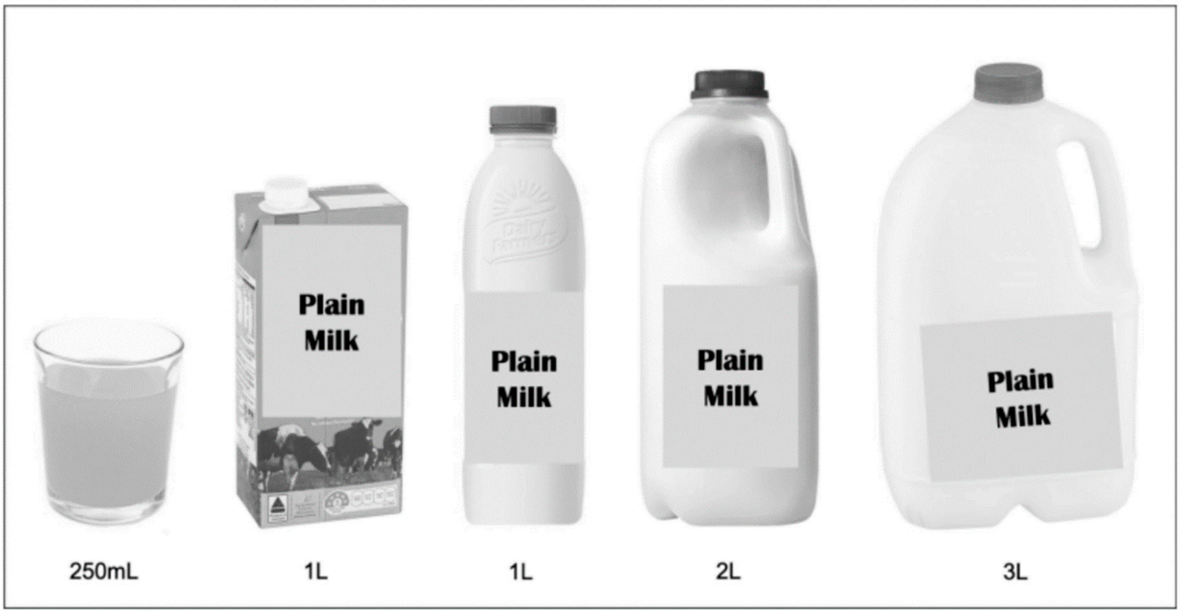

Figure S6. *Flavoured Milk*

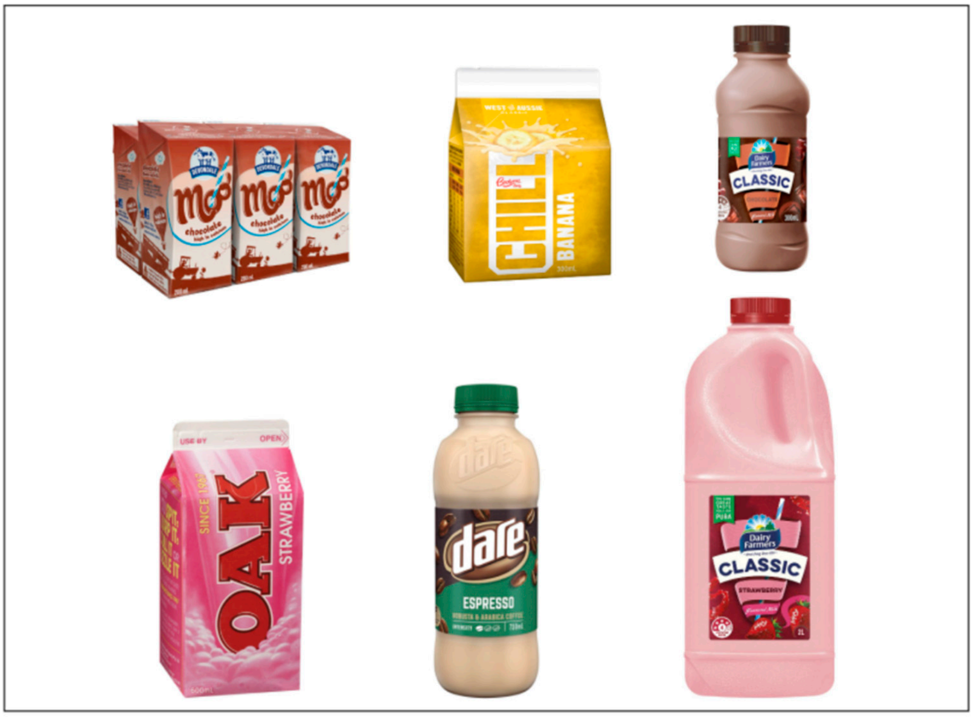

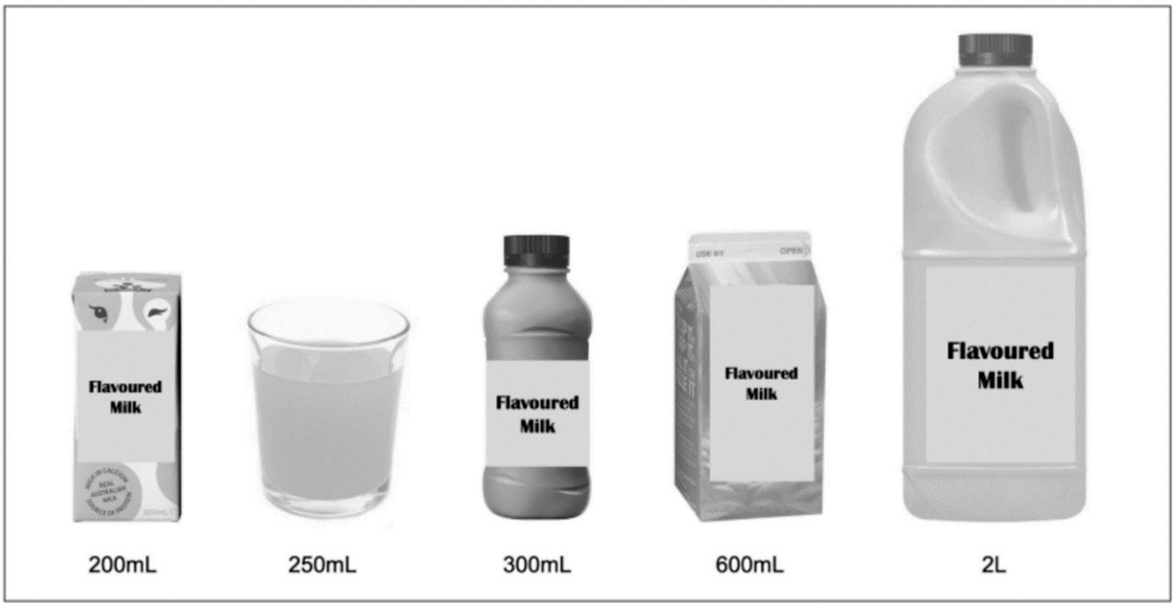

Figure S7. *Tap Water*

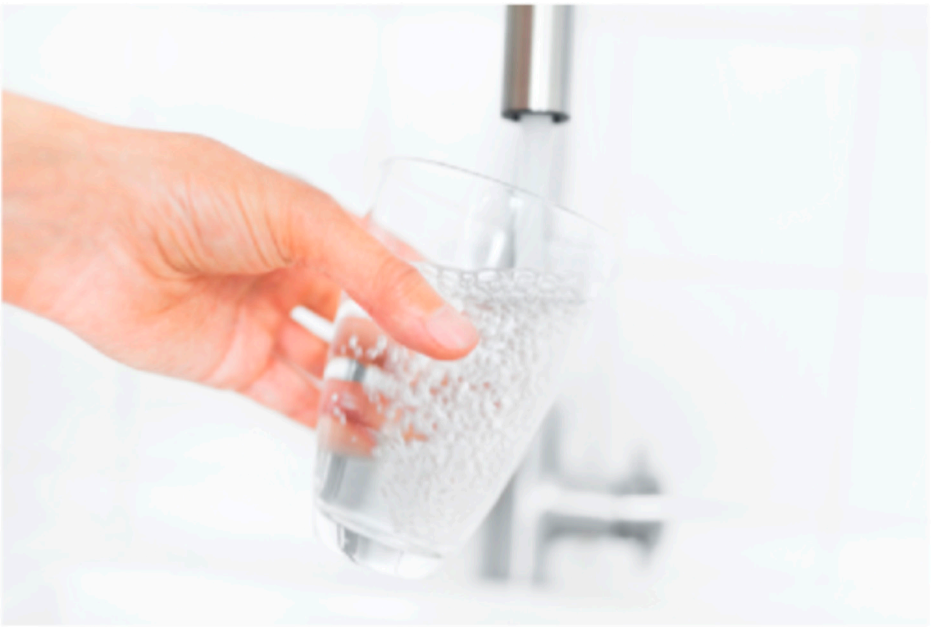

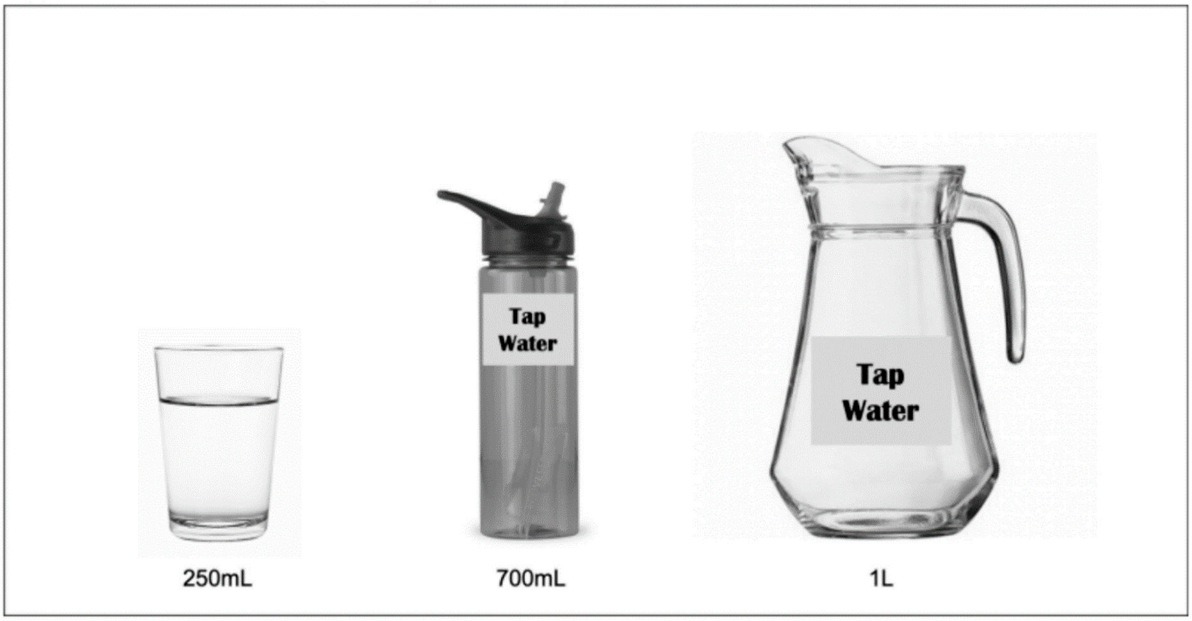

Table S1. *Ninety-fifth Percentiles of Total Volume Consumption for Each Drink Type (ml's)*

Drink Type	Age	Variable 95th Percentile
Soft Drink	Parent	>4620
	Child	>1250
100% Fruit Juice	Parent	>3600
	Child	>2470
Plain Milk	Parent	>9000
	Child	>6000
NNS beverages	Parent	>4430
	Child	>1200
Fruit Drink	Parent	>4000
	Child	>2000
Flavoured Milk	Parent	>2450
	Child	>1800
Tap Water	Parent	>14000
	Child	>14000

Table S2. *Coefficients from Zero-Inflated Regression Analyses Predicting Child Consumption of Each Beverage Type with Perceptions*

		Negative Binomial			Logistic		
Beverage Type	Predictor	Coefficient	Std. Error	*p*	Coefficient	Std. Error	*p*
100% Fruit Juice[a]	Healthy	-0.01	0.016	0.525	0.014	0.037	0.713
	Tasty	0.013	0.019	0.499	-0.142	0.038	< .001
	Good For Kids	0.05	0.017	.003	-0.042	0.04	0.284
	Cheap	-0.013	0.009	0.161	-0.028	0.023	0.229
	Low in Sugar	0.03	0.01	.002	-0.033	0.024	0.178
	Not Okay to Have Everyday	0.015	0.01	0.131	0.135	0.024	< .001
Fruit Drink[b]	Healthy	0.027	0.014	.065	-0.037	0.038	0.336
	Tasty	-0.01	0.016	0.512	-0.072	0.037	.050
	Good For Kids	-0.004	0.016	0.814	-0.063	0.042	0.139
	Cheap	0.016	0.01	0.130	0.019	0.027	0.479
	Low in Sugar	0.01	0.011	0.356	-0.059	0.029	.044
	Not Okay to Have Everyday	-0.001	0.009	0.935	0.117	0.026	< .001
Flavoured Milk	Healthy	-0.004	0.015	0.781	-0.162	0.034	< .001
	Tasty	0.007	0.02	0.735	-0.038	0.039	0.338
	Good For Kids	-0.003	0.018	0.854	-0.055	0.04	0.167
	Cheap	0.005	0.012	0.667	0.02	0.028	0.46
	Low in Sugar	0.004	0.013	0.79	0.002	0.031	0.956
	Not Okay to Have Everyday	-0.022	0.011	0.047	0.076	0.025	0.003
Plain Milk[a]	Healthy	-0.007	0.018	0.674	-0.044	0.048	0.355
	Tasty	0.027	0.011	.013	-0.271	0.028	< .001
	Good For Kids	0.027	0.017	0.117	-0.087	0.048	.069
	Cheap	-0.002	0.008	0.755	0.014	0.028	0.608
	Low in Sugar	0.036	0.01	< .001	-0.003	0.032	0.919
	Not Okay to Have Everyday	0.047	0.009	< .001	0.091	0.027	0.001

Soft Drink[b]	Healthy	0.018	0.014	0.202	-0.122	0.043	.004
	Tasty	0.049	0.016	.002	-0.155	0.039	< .001
	Good For Kids	-0.006	0.015	0.681	-0.014	0.044	0.752
	Cheap	0.002	0.011	0.835	-0.021	0.032	0.527
	Low in Sugar	0.013	0.015	0.397	-0.017	0.045	0.704
	Not Okay to Have Everyday	-0.02	0.01	0.053	0.157	0.032	< .001
NNS Beverages[c]	Healthy	0.014	0.019	0.461	-0.111	0.046	.015
	Tasty	0.02	0.016	0.229	-0.15	0.041	< .001
	Good For Kids	-0.014	0.02	0.483	-0.061	0.05	0.22
	Cheap	-0.001	0.015	0.949	-0.119	0.04	.003
	Low in Sugar	-0.008	0.013	0.55	0.02	0.036	0.572
	Not Okay to Have Everyday	-0.004	0.013	0.744	0.071	0.036	0.048
Tap Water	Healthy	0.065	0.018	< .001	-0.279	0.052	< .001
	Tasty	0.01	0.005	.080	-0.015	0.031	0.623
	Good For Kids	0.004	0.015	0.808	-0.219	0.053	< .001
	Cheap	-0.002	0.008	0.756	0.077	0.046	.091
	Low in Sugar	0.012	0.012	0.295	-0.018	0.05	0.71
	Not Okay to Have Everyday	-0.008	0.008	0.296	0.112	0.03	< .001

[a] Controlled for Parent Education. [b] Controlled for Child Age and Weight. [c] Controlled for Child Age. The logistic component coefficients represent the log odds ratio of consuming 0mL compared to consuming more than 0mL, whereas the zero-inflated negative binomial component coefficients represent the expected change in the log of the outcome (consumption in mL).
